# Predictive Value of Neutrophil-to-Lymphocyte Ratio in Differentiating Perforated from Non-perforated Appendicitis: A Cross-Sectional Study in a Tertiary Care Hospital, Tamil Nadu

**DOI:** 10.7759/cureus.62030

**Published:** 2024-06-09

**Authors:** Gautham Sidharth R, Gautham Gunasekaran, Kishore A, Sujay Jeffrey R, Surendran Paramsivam

**Affiliations:** 1 General Surgery, Sri Ramachandra Institute of Higher Education and Research, Chennai, IND

**Keywords:** forecasting, intestinal perforation, lymphocytes, neutrophils, appendicitis

## Abstract

Introduction

Acute appendicitis is a common reason for acute abdominal pain. It has a high perforation rate of 20%. Diagnosis of acute appendicitis is usually through well-known clinical signs and symptoms. Radiologic imaging is by and large carried out in peculiar cases with indistinct signs and symptoms. Although various scoring methods are available for screening and diagnosis, those have inadequate validity to accurately predict the severity of acute appendicitis. From the differential counts, the neutrophil-to-lymphocyte ratio (NLR) is an economical and straightforward measure of subclinical inflammation. NLR may be a useful marker for predicting the onset and severity of appendicitis because of the insight it gives into immunological and inflammatory pathways. In this study, we aimed to determine the association between NLR and acute appendicitis among adult patients to differentiate between perforated and non-perforated appendicitis in a tertiary care hospital in Tamil Nadu, India.

Methods

This was a cross-sectional study conducted in the Department of General Surgery of a deemed university in Chennai, Tamil Nadu. The study was conducted from March 2022 to December 2022. Patients aged 18 years and above undergoing appendicectomy surgery were included in the study. Patients with hematology disorders, chronic kidney disease, chronic liver disease, chronic obstructive pulmonary disease, asthma, cancer, or auto-immune diseases, and any viral, bacterial, or parasitic infections were excluded. Pregnant women were also excluded from the study. After obtaining informed consent from the patients, blood samples were collected as and when they were diagnosed as acute appendicitis. Laboratory analysis for complete hemogram including white blood cell (WBC) count, neutrophil, and lymphocyte count was carried out using an automated hematology analyzer. Prevalence of perforated appendicitis was reported as a percentage. The receiver-operating characteristic (ROC) curve was developed for NLR in differentiating perforated and non-perforated appendicitis. Data were entered in Microsoft Excel 2023. These analyses were carried out in STATA 12.0 (StataCorp, College Station, Texas, USA).

Results

A total of 212 patients aged 18 years and above were included in the study. Among them 93 (43.9%) were male and 119 (56.1%) were female. Prevalence of perforated appendicitis observed intra-operatively was 29.7% and non-perforated appendicitis was 70.3%. The mean (SD) of NLR among patients with perforated appendicitis was 8.8 (5.1) and non-perforated appendicitis was 3.2 (2.4) with a statistically significant difference (p-value < 0.0001). ROC curve with a cut-off value of 3.78 NLR, had sensitivity of 65.9% and specificity of 93.1% in differentiating perforated and non-perforated appendicitis. The positive predictive value (PPV) and negative predictive values (NPV) were reported as 85.7% and 81.2%, respectively.

Conclusion

NLR has a reasonable validity in differentiating perforated and non-perforated appendicitis. NLR may be useful in low-resource settings where routine confirmatory radiological procedures like computed tomography scans are not available.

## Introduction

Acute appendicitis is a common cause of sudden abdominal pain, with a lifetime occurrence rate of 7%. It has a high rate of perforation, up to 20% [1]. However, the diagnosis of acute appendicitis can be challenging in low-resource settings, despite the presence of well-known symptoms and clinical signs [2]. The diagnosis of acute appendicitis is mainly based on clinical presentation, and radiologic imaging is typically done in specific cases and is not readily available. Delayed diagnosis of acute appendicitis can lead to severe complications such as perforation, which can result in significant morbidity, and even death [3].

Acute appendicitis can be diagnosed with the help of a number of screening and scoring methods, such as the Alvarado score and the Raja Isteri Pengiran Anak Saleha Appendicitis (RIPASA) score [4]. The RIPASA score is a simple scoring technique and consists of covariates such as age, sex, duration of symptoms, Rovsing’s sign, right iliac fossa guarding, and results of urinalysis. However, these scoring techniques have been criticized for not being sensitive or specific enough, and for failing to accurately predict the severity of acute appendicitis [5]. There are also a number of laboratory investigations that may be done to gauge the likelihood of appendicitis and its severity. Although white blood cell (WBC) counts are often increased in individuals with appendicitis, there is no evidence to suggest that this is a reliable predictor of whether or not the condition is simple or complex [6]. Even while an elevated blood bilirubin level might be a sign of appendix perforation, it does not have the necessary sensitivity and specificity. When it comes to predicting perforation in acute appendicitis, C-reactive protein (CRP) is more effective than bilirubin [7]. Discovering a test or marker that can accurately predict an acute appendicitis diagnosis and distinguish between simple and complicated appendicitis is of value for general practitioners in low-resource settings [8].

From the differential WBC counts, the neutrophil-to-lymphocyte ratio (NLR) may be easily determined, making it a cheap and straightforward measure of subclinical inflammation [9]. NLR may be a useful marker for predicting the onset and severity of appendicitis because of the insight it gives into two distinct immunological and inflammatory pathways. The lymphocyte count emphasizes the regulatory pathway, whereas the neutrophil count emphasizes active and ongoing inflammation [10].

In this study, we aimed to determine the association between NLR and acute appendicitis among adult patients to differentiate between perforated and non-perforated appendicitis in a tertiary care hospital in Tamil Nadu, India.

## Materials and methods

This was a cross-sectional study conducted in the Department of General Surgery of a deemed university in Chennai, Tamil Nadu. The study period was from March 2022 to December 2022. All adult patients aged 18 years and above undergoing appendicectomy surgery in the Department of General Surgery were included in the study. Patients with hematological disorders, chronic kidney disease, chronic liver disease, chronic obstructive pulmonary disease, asthma, cancer, or auto-immune diseases, and any viral, bacterial, or parasitic infections were excluded. Pregnant women were also excluded from the study. Blood samples were collected from the patients after obtaining informed consent in writing. The samples were collected as and when they were diagnosed as acute appendicitis.

Laboratory analysis for complete hemogram including WBC count, neutrophil, and lymphocyte count was carried out using automated hematology analyzer. Neutrophil count was determined by multiplying the total WBC count by the percentage of neutrophils present in the differential WBC count [11]. The percentage of neutrophils consists of segmented (mature neutrophils) and bands (almost mature neutrophils).

Neutrophil Count (NC) = [(% segmented neutrophils + Bands) + WBC)]/100)

Normal NC should range from 1.5 to 8.0 (1500-8000 mm^3^). Absolute lymphocyte count (ALC) is the product of the total WBC count and the percentage of lymphocytes.

Absolute Lymphocyte Count = WBC X % lymphocytes

ALC in peripheral blood indicates the body’s immune surveillance potential. The normal range of lymphocytes is typically between 1,300 and 4,000 cells per milliliter. The percentage range should be between 20% and 40% of the WBC count. The NLR was defined as the percentage of neutrophils divided by the percentage of lymphocytes [12].

Data were entered in Microsoft Excel 2023. Prevalence of perforated appendicitis was reported as percentage. Mean (SD) was reported for continuous variables. An unpaired T-test was applied for significance testing of the dependent and independent variables. The receiver-operating characteristic (ROC) curve was developed for NLR in differentiating perforated and non-perforated appendicitis. These analyses were carried out in STATA 12.0 (StataCorp, College Station, Texas, USA).

## Results

A total of 212 patients aged 18 years and above were included in the study. Among them 93 (43.9%) were male and 119 (56.1%) were female. Mean (SD) age of the patients was 37.3 (14.8) years. There is no significant difference in the age and sex distribution of perforated and non-perforated appendicitis. Prevalence of perforated appendicitis observed intra-operatively was 29.7% of patients and the remaining 70.3% (i.e., 149) patients had non-perforated appendicitis. Of the 119 female patients, 31 (49.2%) had perforated appendicitis and 88 (59.1%) had non-perforated appendicitis. Among 93 male patients, 32 (50.8%) had perforated appendicitis and 61 (40.9%) had non-perforated appendicitis.

Mean (SD) neutrophil percent among patients with perforated appendicitis was 80.5 (8.8) and 66.4 (11.8) among non-perforated appendicitis. Mean (SD) lymphocyte percent among patients with perforated appendicitis was 12.4 (7.4) and 26.6 (10.2) percent among patients with non-perforated appendicitis. Mean (SD) of the total count was 12865.4 (5585.1) cells per milliliter and 9457.9 (2557.0) cells per milliliter among perforated and non-perforated appendicitis. Mean (SD) of NLR was 3.2 (1.1). Mean (SD) of NLR among patients with perforated appendicitis was 8.8 (5.1) and non-perforated appendicitis was 3.2 (2.4) with a statistically significant difference (p value < 0.001). There is a statistically significant difference of patients’ laboratory characteristics namely neutrophil percent, lymphocyte percent, total count, and NLR among the patients with perforated and non-perforated appendicitis (Table 1).

**Table 1:** Distribution of perforated and non-perforated appendicitis among various patient characteristics (N=212). The chi-square test was applied for age categories and gender. Unpaired T-test was applied for neutrophil percent, lymphocyte percent, total count, and NLR. SD: standard deviation

Patients’ Characteristics	Perforated Appendicitis (n=63)	Non-perforated Appendicitis (n=149)	Total (n=212)	P Value
Age (years) n (%)				
18–40	39 (69.1)	93 (62.4)	132 (62.3)	0.684
41–59	20 (31.8)	43 (68.3)	63 (29.7)	
60 and above	4 (0.06)	13 (76.5)	17 (0.08)	
Age in years (Mean (SD))	37.7 (14.9)	37.1 (14.7)	37.3 (14.8)	0.809
Gender n (%)				
Female	31 (49.2)	88 (59.1)	119 (56.1)	0.186
Male	32 (50.8)	61 (40.9)	93 (43.9)	
Neutrophil percent (Mean (SD))	80.5 (8.8)	66.4 (11.8)	70.6 (12.8)	<0.001
Lymphocyte percent (Mean (SD))	12.4 (7.4)	26.6 (10.2)	22.4 (11.4)	<0.001
Total count (Mean (SD))	12865.4 (5585.1)	9457.9 (2557.0)	11161.7 (4071.0)	<0.001
NLR (Mean (SD))	8.8 (5.1)	3.2 (2.4)	3.2 (1.1)	<0.001

**Figure 1:**
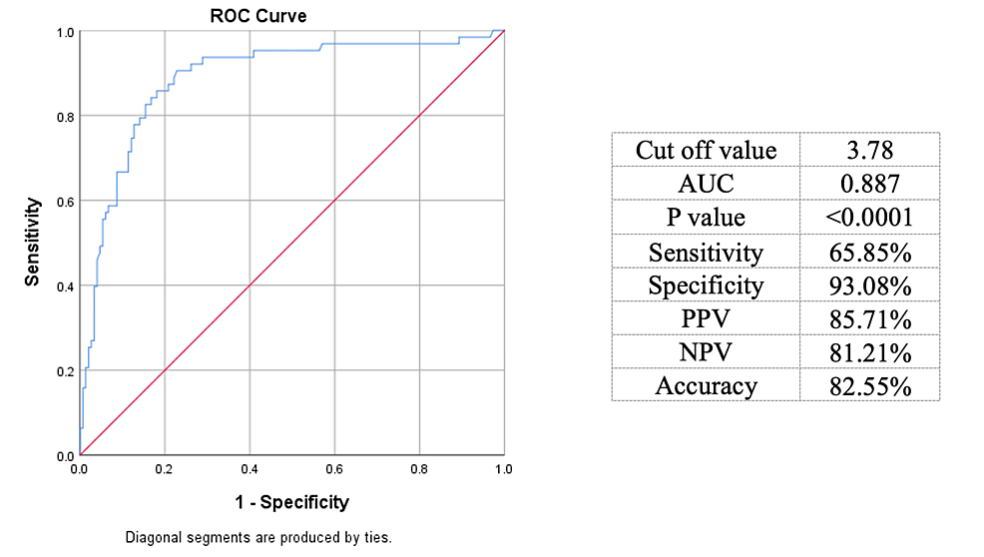
Receiver-operating characteristics of NLR in differentiating perforated and non-perforated appendicitis. NLR: neutrophil-to-lymphocyte ratio; AUC: area under the curve; PPV: positive predictive value; NPV: negative predictive value

ROC curve with a cut-off value of 3.78 NLR had a sensitivity of 65.9% and specificity of 93.1% in differentiating perforated and non-perforated appendicitis. The positive predictive value (PPV) and negative predictive value (NPV) were reported as 85.7% and 81.2%, respectively. The area under the curve (AUC) was 0.887 and accuracy of 82.5% (Figure 1).

## Discussion

This study aimed to evaluate the predictive value of the NLR in differentiating between perforated and non-perforated appendicitis in a tertiary care hospital in Tamil Nadu, India. The findings underscore the significance of NLR as a diagnostic tool in clinical settings where advanced imaging techniques may not be readily available [13,14].

The results align with previous studies that have identified NLR as a valuable marker for inflammatory and infectious conditions [15]. The mean NLR was significantly higher in patients with perforated appendicitis compared to those with non-perforated appendicitis (8.8 vs. 3.2, p < 0.001). This significant difference supports the hypothesis that NLR is elevated in more severe forms of appendicitis due to increased neutrophil counts and decreased lymphocyte counts, reflecting heightened inflammatory response and immune dysregulation [16,17].

The ROC curve revealed a cut-off value of 3.78 for NLR, with a sensitivity of 65.9% and a specificity of 93.1%. These values indicate that while NLR is highly specific in identifying patients with perforated appendicitis, its sensitivity is moderate [13]. The high specificity suggests that NLR is particularly useful in confirming perforated appendicitis when the ratio exceeds the cut-off value. However, the moderate sensitivity implies that some cases of perforated appendicitis might not be detected using NLR alone, necessitating complementary diagnostic methods [18,19].

In resource-limited settings where access to comprehensive imaging modalities like CT scans is restricted, NLR can serve as a cost-effective and readily available marker to aid in the diagnosis and management of acute appendicitis [20]. The PPV of 85.7% and the NPV of 81.2% further enhance its utility, providing clinicians with a reliable tool to stratify patients based on the likelihood of appendiceal perforation [21].

This study’s cross-sectional design is limited by the ability to establish the temporality of the findings. Additionally, the exclusion of patients with various chronic conditions and infections could have influenced the generalizability of the findings [22,23]. However, the study’s strengths include a robust sample size and the use of standardized laboratory methods for measuring NLR, enhancing the reliability of the results.

Further research to validate these findings across different populations and healthcare settings is a requisite. Longitudinal studies could provide deeper insights into the temporal relationship between NLR and the progression of appendicitis. Additionally, exploring the integration of NLR with other inflammatory markers and clinical scores could improve diagnostic accuracy and patient outcomes in resource-limited settings.

## Conclusions

The study concludes that the NLR is a valuable and specific marker for differentiating between perforated and non-perforated appendicitis. NLR in routine clinical practice may aid in timely and accurate diagnosis, potentially reducing the morbidity associated with delayed treatment of perforated appendicitis in low-resource settings.
